# Is Antibody-Dependent Enhancement of *Trypanosoma cruzi* Infection Contributing to Congenital/Neonatal Chagas Disease?

**DOI:** 10.3389/fimmu.2021.723516

**Published:** 2021-09-10

**Authors:** Yves Carlier, Carine Truyens, Eric Muraille

**Affiliations:** ^1^Laboratoire de Parasitologie, Faculté de Médecine, Université Libre de Bruxelles (ULB), Bruxelles, Belgium; ^2^Department of Tropical Medicine, School of Public Health and Tropical Medicine, Tulane University, New Orleans, LA, United States

**Keywords:** *Trypanosoma cruzi*, chagas disease, congenital/neonatal infection, antibodies, FcR, antibody-dependent enhancement

## Abstract

The newborns of women infected with the parasite *Trypanosoma cruzi* (the agent of Chagas disease) can be infected either before birth (congenitally), or after birth (as e.g., by vector route). Congenital Chagas disease can induce high levels of neonatal morbidity and mortality. Parasite-infected pregnant women transmit antibodies to their fetus. Antibodies, by opsonizing parasites, can promote phagocytosis and killing of *T. cruzi* by cells expressing FcγR, on the mandatory condition that such cells are sufficiently activated in an inflammatory context. Antibody-dependent enhancement (ADE) is a mechanism well described in viral infections, by which antibodies enhance entry of infectious agents into host cells by exploiting the phagocytic FcγR pathway. Previously reported Chagas disease studies highlighted a severe reduction of the maternal-fetal/neonatal inflammatory context in parasite-transmitting pregnant women and their congenitally infected newborns. Otherwise, experimental observations brought to light ADE of *T. cruzi* infection (involving FcγR) in mouse pups displaying maternally transferred antibodies, out of an inflammatory context. Herein, based on such data, we discuss the previously unconsidered possibility of a role of ADE in the trans-placental parasite transmission, and/or the development of severe and mortal clinical forms of congenital/neonatal Chagas disease in newborns of *T. cruzi*-infected mothers.

## Introduction

Antibody-dependent enhancement (ADE) is a mechanism by which certain viral infections are enhanced in the presence of antibodies mediating entry of infectious agents into host cells by exploiting the phagocytic FcγR pathway. A lot of studies emphasized the role of ADE in infections with dengue-, zika-, influenza-, Ebola-viruses, HIV, RSV and more recently coronaviruses (reviewed in 1). ADE can occur when non-neutralizing antibodies or antibodies at sub-neutralizing levels bind to viral antigens without blocking or clearing infection. It is particularly relevant in the context of pre-existing immunity, when viruses are in the presence of circulating antibodies resulting from either a previous infection or maternal transmission to neonates. Interestingly, ADE was initially mentioned to explain an increased incidence of dengue hemorrhagic fever in Thai infected infants born to immune mothers that ostensibly had pre-existing antibodies to dengue virus (2, 3).

The ADE concept can be extended to infections with intracellular protozoan parasites and particularly to the infection with the hemoflagellate *Trypanosoma cruzi*. This parasite comes in two forms: the trypomastigote (the free circulating parasite form) and the amastigote (the intracellular multiplication form) (4). It is the agent of the tropical Chagas disease (CD), transmitted in humans by insect vectors, blood transfusion, organ transplantation, orally (by ingestion of food or liquid contaminated with *T. cruzi*), or by maternal-fetal route (5). Congenital CD (CCD) remains an important global and neglected public health problem. In the currently 21 endemic Latin American countries, 1,125,000 women in fertile age are estimated infected with *T. cruzi* (mainly since childhood through insect vector transmission), with an incidence of congenital infection of 8668 cases/year (6). About 5% of pregnant women chronically infected with *T. cruzi* (the most frequent clinical phase of CD) give birth to infected newborns (7). Through migrations of Latin American people, cases of CCD are also reported in North America, Europe, Australia and Japan, where vector transmission does not occur (or is extremely rare) (8). Beside asymptomatic cases, *T. cruzi*-infected newborns can exhibit fever, low birth weight, prematurity, hepatosplenomegaly, pneumonitis, premature rupture of membranes, until presenting pejorative outcomes with high levels of neonatal morbidity (meningoencephalitis, myocarditis) and mortality. Left untreated, the neonatal infection can progress to chronic CD later in life (susceptible to induce myocardiopathy or digestive megaviscera) (reviewed in 9, 10).

Here, based on reported human and experimental data from our team and others, we aim to discuss the previously unconsidered possibility of a role of ADE in the development and/or worsening of CD in neonates born to *T. cruzi*-infected mothers. Such newborns display maternally derived antibodies and can be infected either congenitally (as in endemic and non-endemic areas) or after birth, by another transmission route (as e.g. by vector route as occurring in LA endemic countries).

## Antibody responses in *T. cruzi* infection

Humoral immune response in humans as well as experimental *T. cruzi* infection in mice is diverse and complex, including both parasite-specific and unspecific antibodies (Ab) arising from polyclonal B cell activation. Parasitemia (blood amount of trypomastigotes) starts to decrease when *T. cruzi*-specific Ab reach their highest levels (in the transition from acute to chronic phase of infection), suggesting their protective role (reviewed in 11).

Some Ab have neutralizing functions, e.g. against the glycoproteins protecting the trypomastigotes from the direct action of complement, likewise allowing parasite lysis (12). Others Ab inhibit the activity of major virulence factors of *T. cruzi*, such as the proteolytic enzyme cruzipain. Still other Ab, directed against the parasite trans-sialidases (13), allow the action of anti-α-Gal Ab, another class of lytic Ab, abundantly produced during human infection (14).

Ab, by opsonizing trypomastigotes, can also mediate parasite clearance by promoting their phagocytosis and killing by activated granulocytes, monocytes and macrophages expressing FcγR (15, 16). Such cell activation, resulting from intracellular signals transduced upon FcγR crosslinking and interactions between cytokine(s) and their membrane receptor(s), promotes the generation of reactive nitrogen species able to kill extracellular *T. cruzi* (17).

IgG Ab appear in acute infection and persist life-long during the chronic infection. IgG1-, followed by IgG3-Ab isotypes are mainly produced in human infection, whereas IgG2a-, IgG2b- and IgG1-Ab are more frequently observed in mouse infection, all isotypes being able to bind cell FcγR (18, 19).

## Vertical Transmission of *T. cruzi*-specific Ab

There is a fundamental difference between man and mouse regarding the transfer of maternal IgG to offspring. It occurs through the placenta (from the 13-14^th^ week of pregnancy to delivery) in humans, and by breast milk in mice (from birth to the weaning on day 21 after birth). Such transfer is carried out by transcytosis through a FcRn localized on the membrane of trophoblastic cells in humans or digestive tube cells in mice (20). Most IgG, whatever their Fab specificities are transferred, though some isotypes are preferentially transmitted, due to higher affinities between their Fc portion and the FcRn (21).

In human infection with *T. cruzi*, the trans-placental transmission of Ab occurs as in other infections (22–25). Although few studies have compared the features of transferred antibodies in infected and uninfected neonates from infected mothers, their amounts and repertoires appear similar in infected mothers and their uninfected neonates at birth (23), whereas congenitally infected newborns seem display lower Ab levels than their mothers (26). Such transferred Ab persist up to 8-9 months after birth before becoming undetectable (10, 27).

In murine infection with *T. cruzi*, maternal-offspring transfer of Ab occurs through lactation (28–30), but direct kinetic studies of such transfer are lacking. Our report performed with uninfected mice having received purified *T. cruzi*-specific Ab during gestation and lactation period, showed that Ab levels were reduced by 4 times in offspring at the weaning time (i.e. 3 weeks after birth), compared to the maternal Ab level (30). Other infection models indicate that such transferred Ab can persist more than 6 weeks after birth, i.e. more than 3 weeks after weaning in mouse pups (31).

## Experimental Evidences of Ade of *T. cruzi* infection in Mouse Pups Displaying Maternally Transferred Ab

As shown in various experiments, whatever the used *T. cruzi* genotype/strain, acute infection in mice either prevents gestation or, in case of gestation development, induces high pup mortality (probably related to the inflammatory storm produced by the parasite inoculation) with rare congenital infection in the few surviving pups. When gestation occurs during chronic infection, congenital transmission is not observed, even after looking for cryptic infection in pups immunosuppressed by cyclophosphamide (32, 33).

The mouse model being not suitable for studying *T. cruzi* congenital infection, studies were performed using offspring (uninfected) born to chronically infected or uninfected dams, and experimentally infected after weaning (on two months after birth). Higher parasitemia and mortality rates were observed in offspring of infected mice compared with control offspring. The most severe infections were noted when offspring were born and suckled by their mothers, but no more when offspring was infected 5 (instead of 2) months after birth. Such effect was not seen if offspring was infected with *Plasmodium chabaudi* or *Schistosoma mansoni*. Such results highlight a maternally induced *T. cruzi*-specific enhancement of infection in offspring of infected dams (34). However, the mechanism of such enhancement and the nature of what was transferred from dams to pups to induce it remained unclear.

In order to study more specifically the role of Ab in the previously demonstrated enhancement of post-natal infection in progeny of infected mice, another experiment was performed by injecting either serum from chronically infected animals or purified *T. cruzi*-specific Ab into uninfected mice (born to uninfected dams) during gestation and lactation periods. It was verified that injected Ab were transferred to offspring. When infected two months after birth (as in the experiments mentioned above), offspring of mice treated with chronic serum or purified Ab displayed significantly higher parasitemia and mortality rates than offspring from mothers receiving control serum or immunoglobulins unrelated to *T. cruzi* (30). These results indicate an ADE-like phenomenon in experimental *T. cruzi* infection, and particularly in mice receiving maternally derived Ab.

## Experimental Evidences of a Fcγr-Involvement in Ade of *T. cruzi* Infection in Mice

Flow cytometry studies were performed in mice using the 2.4G2 monoclonal Ab (MoAb), specific to the extracellular domains of low-affinity Fc receptors for IgG (FcγRII/III, CD31 and CD16 respectively). Membrane of splenic and mesenteric lymph nodes cells of *T. cruzi*-infected mice displayed higher expression and absolute number of FcγR in the early and late parasitemic phase (before and after the rise of Ab), compared to uninfected mice (35).

In order to investigate the role of such FcγR, the 2.4G2 MoAb was injected into mice (2 months old). Repeated injections every 3-4 days decreased the availability of FcγR on peritoneal, lymph node, and spleen cells in control uninfected mice. Injections of 2.4G2 MoAb just before parasite inoculation and during the acute phase of *T. cruzi* infection strongly reduced mortality and parasitemia in comparison to control animals receiving an unrelated MoAb, whereas the levels of immunoglobulins and/or *T. cruzi*-specific Ab remained similar in infected and control mice (36).

These results, associated with those mentioned above, indicate that FcγR play a role in the ADE-like phenomenon observed in mice infected with *T. cruzi*. They confirm previous *in vitro* experiments showing the role of parasite-opsonizing Ab in the enhancement of cell parasitic infection (37), besides the various other mechanisms allowing invasion of phagocytic and non-phagocytic host cells by the infective trypomastigote form of *T. cruzi* (38).

## Pregnancy/Gestation, Placental and Neonatal Inflammasomes During *T. cruzi* infection

It is important to note that in the experiments mentioned above (30), apart the Ab, no inflammatory- or other immunological-components were transferred to the offspring. Moreover, there was neither placental inflammation, nor other disturbances likely to be a source of interference in offspring, since dams were uninfected. This suggests that the absence or, at least, a severe reduction of the maternally-derived inflammatory environment might be a key factor promoting ADE by limiting in offspring the cell activation necessary to the protective role of parasite opsonizing Ab (see above). This raises the question of the maternal/fetal/neonatal inflammatory status in case of *T. cruzi* infection.

Although few studies were performed in experimental models, it was shown that gestation associated with acute or chronic *T. cruzi* infection in mice promotes a strong inflammatory response (32, 39, 40), and that an inflammatory status induced in mother rat contributes to the control of *T. cruzi* infection in offspring (41).

The first and third trimesters of human pregnancy are known to induce a strong physiological inflammation status (42). Recent studies showed that the second trimester of pregnancy also induces inflammasome signaling in placental trophoblasts to promote fetal and maternal antimicrobial defenses (43).

Infection during pregnancy increases still more such inflammatory context. Indeed, *T. cruzi* **-**infected pregnant women display a hyperactivation of blood cells releasing various pro-inflammatory cytokines such as IL-1β, IL-6, TNF-α and IFN-γ, in response to *T. cruzi* or LPS/PHA, compared to non-infected pregnant women (44, 45). IFN-γ is the key cytokine controlling *T. cruzi* infection, as other intracellular pathogens. It activates monocytes/macrophages and stimulates, in synergy with TNF-α, the generation of NO which kills parasites (11, 17). However, there is an important difference in the inflammatory status, between pregnant women having given birth to either infected or uninfected neonates. Cells from transmitting pregnant women are phenotypically less activated, and produce less inflammatory cytokines compared to mothers of uninfected newborns (44, 46, 47). Indeed, cells of such mothers produce 3 times less IFN-γ when stimulated with *T. cruzi*, and such defective response persist after delivery. The observation that mothers of infected newborns harbor higher parasitemia than those delivering uninfected babies (9, 47, 48), argues still more for the lower inflammatory capacity of these firsts.

Interestingly, the inflammatory status observed in infected pregnant women is also observed in their neonates (maternal imprinting). Uninfected neonates of infected mothers display leukocytes releasing more TNF-α and higher circulating levels of TNF-α, IFN-γ and IL-18 compared to infected neonates (49, 50). They also present a higher proportion of CD56^bright^ NK cells producing IFN-γ (44, 51). Such Th1 pro-inflammatory status persisted in infant life, with a promoting effect on responses to vaccines (52).

Altogether, such results indicate that: i) a stronger inflammatory state than in normal gestation is induced on both sides of placenta when gestation is associated to acute or chronic infection with *T. cruzi* (in mouse and human infection); ii) such inflammatory context is strongly reduced in case of congenital parasite transmission (in human CCD).

## Might Maternal Ab Enhance Trans-Placental Transmission of Parasites in Humans?

CCD results from a two step process: i) the parasite transmission from mother to fetus through the placenta, and, ii) the development of parasitic infection in the fetus/neonate (multiplication of parasites resulting in CCD more or less severe, see above). Both steps depend on specific mechanisms detailed elsewhere (9, 53, 54). However, as far as we know, the possible enhancing role of Ab present in maternal blood, in the trans-placental transmission of parasites through the trophoblastic FcRn, has not been studied. Interestingly, it has been reported that human cytomegalovirus co-opt FcRn-mediated transcytosis and are transported across syncytiotrophoblasts in immune complexes that infect underlying cytotrophoblasts and are captured by macrophages in the villus core (55, 56). If this is a plausible mechanism also for *T. cruzi* is worth questioning.

This might occur only if parasites are observed in trophoblast and placental tissues (histopathological studies). Such cases are encountered only when very high maternal parasitemia have overwhelmed the placental capacity of defenses, resulting in abortions or delivery of neonates with severe CCD. By contrast, this is unlikely in pregnant women displaying moderated blood parasite amounts (the most frequent case nowadays), since the activation of placental innate defenses (and particularly the trophoblast turnover) is sufficient enough to prevent the trophoblast crossing. In such cases, the parasite population remaining present into the intervillous space, infects the epithelial cells of the marginal zone deprived of trophoblast to be transferred into the fetal blood vessels (9, 53, 54).

The fact that congenital infection is almost absent in mice in which there is neither trophoblastic FcRn nor subsequent trans-placental Ab transfer (see above), might argue for a possible role of the trophoblastic FcRn in the transmission of Ab-opsonized parasites in human infection,

## Might ADE Contribute to the Severity of Human Neonatal CD?

Clinical surveys in endemic areas have clearly shown that severe and lethal CCD are related to high parasitemia in newborns (53, 57, 58).

Indeed, data mentioned above indicate that:

i) Besides their capacity to neutralize essential parasite molecules, Ab, by opsonizing parasites, can promote their phagocytosis and killing by cells expressing FcγR on the mandatory condition that such cells are sufficiently activated in an inflammatory context;
ii) The maternal-fetal/neonatal inflammatory context is seriously reduced in the 5% of infected and parasite-transmitting pregnant women, and their congenitally infected newborns;
iii) The levels of transmitted Ab seems lower in congenitally infected neonates than in uninfected newborns delivered by infected mothers;
iv) Experiments in mice clearly indicate the occurrence of ADE of *T. cruzi* infection, particularly when parasites are in presence of low levels of transmitted maternal Ab, outside of an inflammatory context.

**Figure 1:**
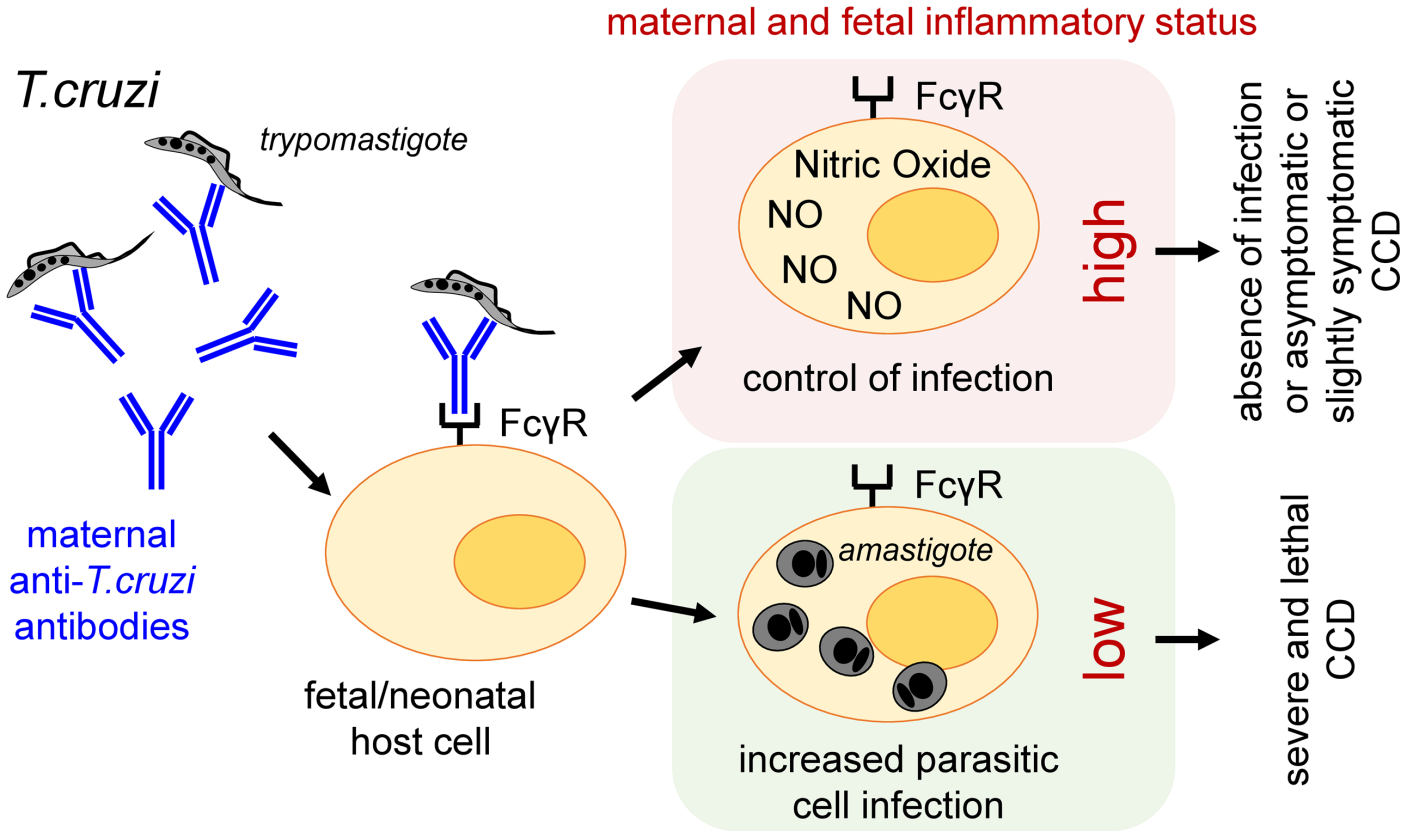
Proposed role of ADE in the development and/or worsening of CCD in humans.

Would be the observations in mice extrapolable to human? The question raised here concerns the potential role of ADE in neonates infected either before- (congenitally) or after-birth (by another transmission route), both having previously received maternally transmitted Ab. In other words, might low amounts of Ab (likely insufficient to sustain a frank protective activity) opsonize circulating parasites and favor their entry into FcγR expressing cells not sufficiently activated to trigger their protective killing burst? Might such enhancement of their intracellular multiplication (leading to high parasite load), worsen infection and lead to more severe and lethal clinical forms of CCD, in some infected newborns (**Figure 1**)? Indeed, such severe forms, though less frequent nowadays, are always observed in endemic as well as non-endemic areas of CD [see below; (9)].

## Possible Factors Biasing Host Towards ADE

Internal as well as external factors might drive towards ADE.

Beside the weakness of the inflammatory context and the global availability of Ab, other intrinsic individual factors might contribute to ADE, such as the distribution of IgG isotypes (presenting different affinities for FcγR) among available Ab, or the allelic distribution of FcγR on host cells (1). Information is lacking on qualitative or quantitative differences in maternal Ab isotype population transmitted in uninfected vs infected babies and between the severity gradients of CCD among infected ones. The allelic distribution of FcγR in human population is not homogenous and some variants do not allow cell activation. Whether such a possibility would condition ADE in *T. cruzi* infection remains to be investigated by characterizing the Fc domain structure and allelic distribution of FcγR genetic variants in neonates of infected mothers. Indeed, epidemiological elements suggest that genetic traits might be, at least partially, related to CCD, such as the familial clustering of congenital cases and the repetition of congenital transmission in successive pregnancies in the same women (59, 60), as well the persistent lower capacity of cells of such women to produce IFN-γ (47).

In addition, other external factors, such as malnutrition and poverty, by affecting the features of innate immune response with cytokine patterns skewed towards a Th2-response, and reducing the Ab production (61), might contribute to favor ADE of *T. cruzi* infection. In line with this possibility, are the reported observations on the progressive decrease of severity of CCD overtime, which was much more severe and deadly in the past when malnutrition and poverty was particularly important in endemic LA countries (9). For instance, we reported mortality rates up to 13% in a Bolivian cohort of congenitally infected newborns studied between 1992 and 1994, while such rate dropped to 2% in another study in 1999-2001, when Bolivia benefited from improved socio-economic conditions and better maternal care (62).

## Conclusions And Perspectives

Although the ADE concept was initially described in viral infections, it has been also shown in bacterial infections and diseases (likely with distinct underlying mechanisms; reviewed in 63). Its extension to protozoan parasite would open new perspectives on the balance between protective and enhancing/facilitating Ab in the immunological homeostasis in response to infections.

Data from clinical surveys, combined with experimental observations, allow us to propose the working hypothesis of a role of ADE in the trans-placental parasite transmission, and/or the development of particularly severe and mortal clinical forms of congenital/neonatal CD. Such amplification of fetal or neonatal infection, through the phagocytic FcγR pathway, in fetuses/newborns having previously received antibodies from their mothers, might contribute to explain the more detrimental pole of the large clinical spectrum of CCD, as well as its historical evolution.

The balance between ADE and Ab-protective responses might depend on the maternal-fetal/neonatal relationship, and particularly on the features of the Ab populations and inflammatory context (with a possible inverse correlation between ADE and the maternal/fetal/neonatal inflammatory context), likely associated to other external or genetic factors. This working hypothesis should stimulate further research works exploring the potential role of FcRn and ADE in the trans-placental transmission of parasites in humans, using *ex vivo* infection of placental explants or trophoblastic cell lines with opsonized *T. cruzi* parasites, or, perhaps also, humanized mice expressing placental FcRn. Other investigations would be particularly welcome on Ab isotype concentrations, inflammation markers, and allelic distribution of cellular FcγR in *T. cruzi*-infected mothers and their neonates, in order to determine the conditions favoring ADE. A better knowledge of conditions contributing to the severity of human neonatal CD might lead to improvements of our diagnosis tools to establish a prognosis of more severe CCD, and focus more attention on such risky group of pregnant women (9).

## Data Availability Statement

The original contributions presented in the study are included in the article/supplementary files. Further inquiries can be directed to the corresponding author.

## Author Contributions

YC sustained the working hypothesis which was discussed with EM and CT. YC wrote the manuscript. All authors contributed to the article and approved the submitted version.

## Conflict of Interest

The authors declare that the research was conducted in the absence of any commercial or financial relationships that could be construed as a potential conflict of interest.

## Publisher’s Note

All claims expressed in this article are solely those of the authors and do not necessarily represent those of their affiliated organizations, or those of the publisher, the editors and the reviewers. Any product that may be evaluated in this article, or claim that may be made by its manufacturer, is not guaranteed or endorsed by the publisher.
